# Brief Report: Preferred Processing of Social Stimuli in Autism: A Perception Task

**DOI:** 10.1007/s10803-021-05195-2

**Published:** 2021-09-16

**Authors:** A. Meermeier, M. Jording, Y. Alayoubi, David H. V. Vogel, K. Vogeley, R. Tepest

**Affiliations:** 1 University Hospital Cologne, NRW, Kerpener Strasse 62, Geb. 31, 50931 Cologne, Germany; 2 Forschungszentrum Jülich, INM3, NRW, Wilhelm-Johnen-Straße 1, 52428 Jülich, Germany

**Keywords:** Social stimuli, Perception task, Autism spectrum disorder, Image recognition, Image persistence

## Abstract

In this study we investigate whether persons with autism spectrum disorder (ASD) perceive social images differently than control participants (CON) in a graded perception task in which stimuli emerged from noise before dissipating into noise again. We presented either social stimuli (humans) or non-social stimuli (objects or animals). ASD were slower to recognize images during their emergence, but as fast as CON when indicating the dissipation of the image irrespective of its content. Social stimuli were recognized faster and remained discernable longer in both diagnostic groups. Thus, ASD participants show a largely intact preference for the processing of social images. An exploratory analysis of response subsets reveals subtle differences between groups that could be investigated in future studies.

The question whether persons with autism spectrum disorder (ASD) experience social stimuli differently than control persons without ASD (CON) has been widely debated. In non-autistic persons there is a very stable preference for processing social stimuli (depicting humans, i.e. faces and body parts), either reflected in shorter reaction times in a detection task for faces and body parts (Ro et al., 2007) or a better detection or discrimination of social stimuli (Bruce et al., 1991; Kikuchi et al., 2009; Lehky, 2000; Ro et al., 2001). Briefly presented faces are detected faster and more accurately than objects (Purcell & Stewart, 1988) and salient social stimuli (i.e. upright faces) can get access to consciousness faster than less salient stimuli (i.e. upside-down faces, Jiang et al., 2007).

In ASD visual attention towards social stimuli seems to be decreased compared to control persons (Chita-Tegmark, 2016; Frazier et al., 2017). Changes in social stimuli were not faster detected than changes in object stimuli by children with ASD in contrast to their healthy peers (Kikuchi et al., 2009). Social stimuli in comparison to non-social stimuli interfere more with a Stroop-task for typically developing children in contrast to children with ASD (Chevallier et al., 2013). Impairments of the ability to recognize faces have been observed in persons with ASD, but here results were less consistent and partially even contradictory (Guillon et al., 2016; Tang et al., 2015; Weigelt et al., 2013). When presented with social stimuli for very brief time spans persons with ASD tended to report less accurately on the gist of the scene (Vanmarcke et al., 2016a) or the kind of interaction (Vanmarcke et al., 2016b) compared to control persons while no differences were observable for non-social stimuli.

However, the question remains, whether these behavioral differences associated with ASD result from a decreased preference for processing and thus recognition of social stimuli or from correct recognition but less relevance attribution and thus attention towards social stimuli in ASD or both.

To investigate the ability to recognize visual stimuli in ASD and to test whether social stimuli are processed preferentially in ASD we have constructed two different perception tasks. In the first task, we investigated how quickly individuals with and without ASD accurately recognize social and non-social images appearing from noise. Therefore, we presented stimuli depicting mere noise gradually resolving over 8 s, revealing either social (containing one or several humans) or non-social scenes (objects or animals). All stimuli were created so that potential systematic differences in low-level image features between social and non-social images were reduced to a minimum. We assessed at which point in time – corresponding to different levels of noise – participants correctly recognized the scene. We expected that social stimuli would be recognized earlier than non-social stimuli when emerging from noise. We did not expect any differences between the two diagnostic groups in their mean recognition times, but the difference in recognition times between social and non-social stimuli was hypothesized to be less pronounced in persons with ASD.

In a second task, images disappeared into noise (i.e., gradually turning from a recognizable image into noise over 8 s) and participants had to report the point in time at which they were no longer able to recognize the image anymore. This task allowed us to rule out influences of potential differences in response times in participants with ASD and to control for potential effects of stimulus familiarity and stimulus heterogeneity. Given the high relevance of social information in everyday life, we predicted a generally more stable persistence of social images in comparison to non-social images, resulting in a later response for social stimuli. We expected that this difference in stability of image types was less pronounced in persons with ASD compared to control persons without ASD.

As previous studies suggested a mediating effect of the complexity of presented stimuli (Chita-Tegmark, 2016; Frazier et al., 2017; Guillon et al., 2014; Hamilton, 2016), we further explored potential shifts in the effects between images recognized earlier compared to those which were recognized later.

## Methods

### Stimuli

**Fig. 1:**
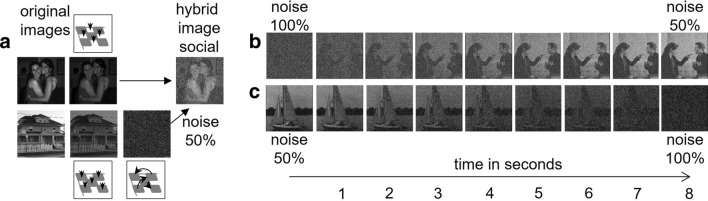
Illustration of the algorithm. **a** A pair of images (social, non-social) is selected. Both images lose half of their informative pixels in a checkerboard- style, to produce a hybrid image. The pixel values of one image (here: the house) are randomized before being combined with pixels of the other image (here: two persons). In the social hybrid image, the information of the social image is preserved whereas the object information of the non-social image is lost. The transformation from the image displaying the house to the image with the random pattern is achieved by spatially re-arranging of the pixels. Please note, that both mean gray values and graytone histograms of all images remain the same. **b** Systematic “emergence” of a social image from noise in the recognition task over 8 s. **c** Systematic “dissipation” of a non-social image into noise in the persistence task

A subset of 120 images from de la Rosa et al. (2014) was rated by 33 participants (18 female, M_age_ 26 years, sd = 5.6 years) in an online study on the perceived degree of social content. Participants rated whether objects or persons were more prominent on a 5-point Likert scale (1 = object to 5 = person). The 25 highest and lowest scoring images were selected as “social” (M_score_ 4.94, sd = 0.09) or respectively “non-social” (M_score_ 1.20, sd = 0.30) and merged to hybrid images by an in-house developed algorithm (based on MATLAB (R2017a, MathWorks®, Natick, MA). The pixels in each case were extracted in an alternating manner from two original images of the different categories. Then the extracted pixels were merged and arranged in a checkerboard order, resulting in hybrid images that combine 50% original pixels each from images of the two categories. As pixels of the initial images are merged in an alternating manner, the recognizability of one image simultaneously disturbs the recognizability of the other one. However, it is possible to control each portion separately and specifically because of the underlying regular checkerboard pattern. A systematic rearrangement of the pixel values of the one or the other source allows a controlled vanishing by stepwise randomization or respectively re-emergence of either the one or the other image. Hence, either the image from the social or the non-social category (Fig. 1) emerged.

It is important to note that only the spatial arrangement of the pixel values is changed. Any change of pixel locations modifies the recognizable content, whereas the mean gray values of the images as a whole and gray value histograms do not change.

### Participants

26 persons without a diagnosis of ASD (CON; M_age_ = 34.15 years, sd = 10.89 years, 13 female) and 23 persons with ASD (ASD; M_age_ = 41.26 years, sd = 8.93 years, 7 female) diagnosed at the outpatient clinic of the University Hospital Cologne participated in the experiment.

Participants with ASD were screened with common self-report instruments (AQ, EQ see Table 1), invited to 2–3 clinical interviews, conducted by at least 2 different professional clinicians according to the criteria of the WHO (ICD-10). A prerequisite for participation in the experiment was the exclusion of learning disabilities or impairments of intelligence (IQ >  = 85, (M = 111.82, SD = 18.15) or qualification/degree from university), and the exclusion of clinical depression (BDI > 19, see Table 1) to avoid any distortions of cognitive processes required by the task instructions. All participants with ASD were fully verbal autistic adults. The sample size was limited by the number of persons with ASD, that were willing to participate and fulfilled the inclusion criteria. Education years did not differ between both groups (see Table 1). We chose person-first language to designate the participants in the study (Tepest, 2021). Groups did systematically differ in age (t-test for independent samples, t_(47)_ = 2.51, p = 0.02).

**Table 1:** Demographic and questionnaire variables including age, education years, AQ, EQ, BDI values

	CON	ASD	t	p
Age (years)	34.153 (10.894)	41.625 (8.930)	2.507	.016*
Education (years)	17.02 (4.912)	17.864 (4.215)	0.63	0.53
AQ values	12.885 (4.982)	39.609 (5.366)	18.0	< .001***
EQ values	44.68 (9.182)	16.391 (8.574)	− 11.037	< .001***
BDI values	3.72 (3.518)	7.0 (5.394)	2.473	.0181*

Group means and standard deviations are reported, including t-values and significance levels (. < .1, * < .05, ** < .01, *** < .001)

### Procedure

Participants underwent the recognition task first, followed by the persistence task. Each stimulus video was presented once per task, resulting in 100 trials per participant. Stimuli (resolution of 300*300 pixel) were presented centrally on a 23″ monitor, participants were seated at approximate 60 cm distance for an image size of 7.6*7.6 degrees visual angle.

### Recognition Task

Starting with a hybrid image with all pixels shuffled (100% noise), the pixels from one of the two composite images were subsequently re-arranged letting this image emerge over the course of 8 s (Fig. 1b). The participants were instructed to press the space key as soon as they were aware of what was depicted in the image. The image would then disappear. To ensure task compliance, the participants were asked to indicate the stimulus content via button press (1 = one person, 2 = several persons, 3 = animal, 4 = inanimate object).

### Persistence Task

Trials started with hybrid images with 50% noise (i.e., randomly shuffled pixels of one of the two composite images, the other of the two composite images being arranged correctly). Subsequently, the correctly ordered pixels were shuffled incrementally over the course of 8 s, too, so that the visible image dissipated into noise (see Fig. 1c). During the increase in noise participants were instructed to indicate via space-key press the moment in time when the image became unrecognizable.

### Analysis

We used R (R Core Team, 2012) and lme4 (Bates et al., 2012) to perform a linear mixed effects analysis of the relationship between social and non-social images and response time in the two different perception tasks for both diagnostic groups. As fixed effects, we entered group (ASD vs. CON) and image type (social vs. non-social) with interaction-term into the model. As random effect, we included intercepts for subjects. Visual inspection of residual plots did not reveal any obvious deviations from homoscedasticity or normality. P-values were obtained by likelihood ratio tests of the full model with the effect in question against the model without the effect in question. Estimates are reported as means and standard errors. As an approximation for effect sizes we report delta total, d_t_ (Brysbaert & Stevens, 2018; Westfall et al., 2014).

Starting with 53 participants we excluded data from one control person who had an AQ value above the threshold of 32. Furthermore, we excluded data from two participants with ASD who had a BDI score above the threshold of 19, resulting in 49 participants (ASD: n = 23; CON: n = 26). All differences between groups were calculated using glm with Poisson link functions. We excluded trials in which no press of the space-key was recorded as missing data (recognition task: ASD 4.96%, CON 2.77%, z = − 2.84 p = 0.004; persistence task: ASD 1.22%, CON = 0.08%, z = − 2.67, p = 0.01). To account for accidental key presses, response times below one second were excluded from analysis (recognition task: ASD 0.0%, CON 0.08, z = 0.003, p = 0.99; persistence task: ASD 0.09%, CON 0.23%, z = 0.84, p = 0.40). Furthermore, we excluded inaccurately categorized trials from the recognition task (ASD 1.91%, CON 1.31%, z = − 1.18, p = 0.24). Finally, we excluded individual outliers, which were defined as recognition or persistence times that were above or below 2 standard deviations of the participant´s individual average in that task (recognition task: ASD 3.74%, CON 4.54%, z = 0.97, p = 0.33; persistence task: ASD 4.52%, CON 3.62%, z = − 1.21, p = 0.226).

### Exploratory Analysis

To investigate whether the degree of complexity of images had the same effect on recognition in the social and non-social categories we ranked the stimuli according to their average response time and split them into an “early responses”(12 stimuli) and a “late responses” (13 stimuli) subset (average difference 1.557 s, se = 0.08 s). The ranking was highly consistent in both groups, resulting in high correlations of average recognition times per stimulus between diagnostic groups for social (r = 0.982 [0.959; 0.992], t_(23)_ = 25.013, p < 0.001) and non-social stimuli (r = 0.951 [0.889; 0.978], t_(23)_ = 14.675, p < 0.001). The effect of response subsets and its interactions with other factors were tested in likelihood ratio tests of linear models. Subsequently, we also compared the recognition response times of the diagnostic groups quantile-wise for the different levels of complexity via the shift-function of the rogme package (Rousselet & Wilcox, 2020).

## Results

### Effects of Age and Gender

To investigate possible effects of age and gender we analyzed both diagnostic groups separately. Neither in the CON group nor the ASD group any of the factors age and gender showed an increased model fit in any of the two tasks [CON recognition task: age (χ^2^_(1)_ = 0.503, p = 0.50), gender (χ^2^_(1)_ = 1.618, p = 0.20); ASD recognition task: age (χ^2^_(1)_ = 0.505, p = 0.48), gender (χ^2^_(1)_ = 0.749, p = 0.39); CON persistence task: age (χ^2^_(1)_ = 0.704, p = 0.40), gender (χ^2^_(1)_ = 0.023, p = 0.88); ASD persistence task: age (χ^2^_(1)_ = 0.001, p = 0.98), gender χ^2^_(1)_ = 2.117, p = 0.15)]. Hence, we found no support for influences of age or gender onto recognition or persistence times.

### Recognition Task

**Fig. 2:**
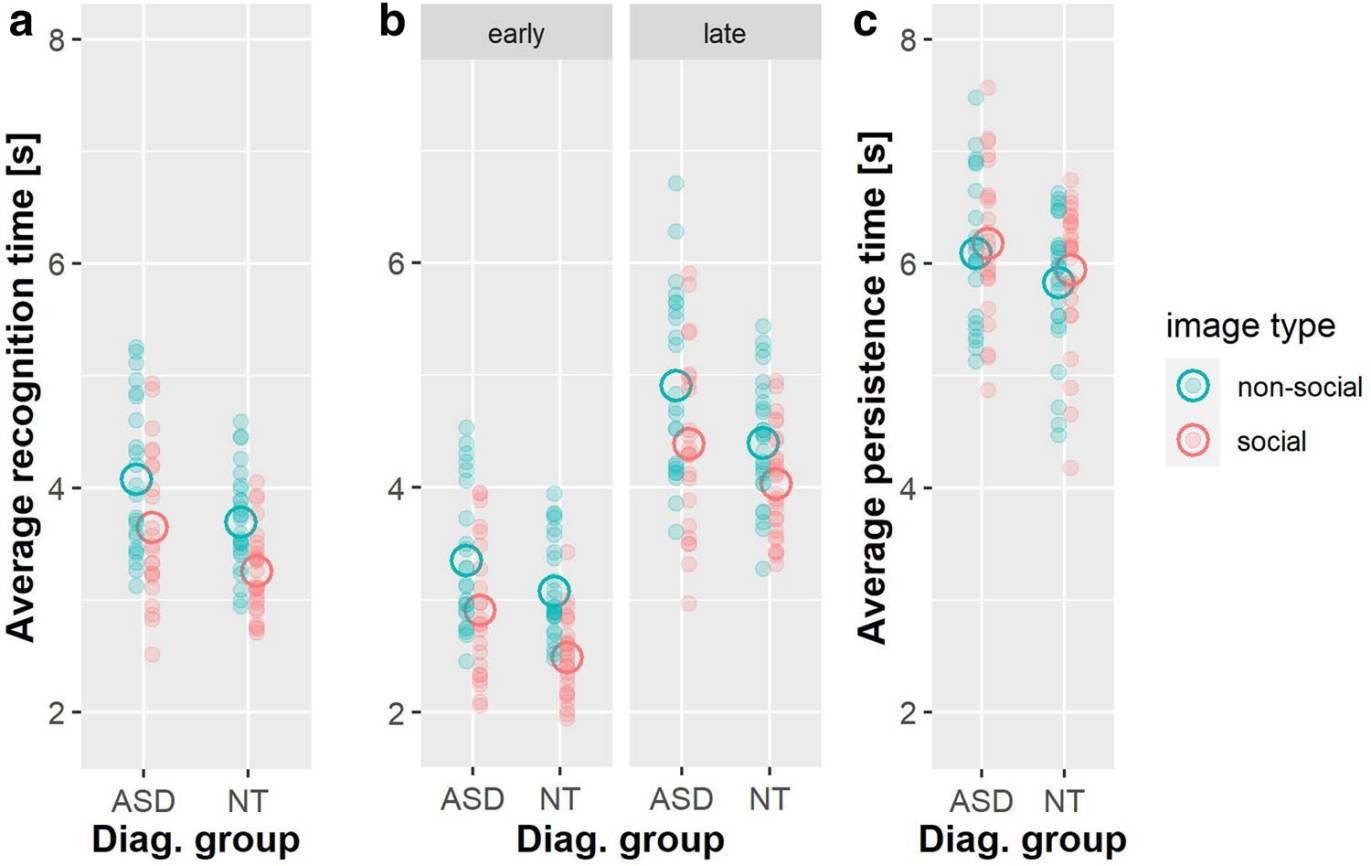
Response times across diagnostic groups and image type during “emergence” of the images. Individual participant’s average response times are depicted by small, filled circles, group averages are depicted by big open circles. Responses on social images are shown in red, responses on non-social ones in blue. **a** Recognition times for the complete data set. **b** Recognition times split into two subsets of early responses and late responses. **c** Persistence times during “dissipation” back into noise. Persistence times for the complete data set

Belonging to group ASD significantly increased the recognition time by 0.382 s (se = 0.16 s) (χ^2^_(1)_ = 6.365, p = 0.012, d_t_ = − 0.299 [− 0.543; − 0.056]). Social stimuli significantly lowered recognition time by 0.423 s (se = 0.07 s), (χ^2^_(1)_ = 74.760, p < 0.001, d_t_ = − 0.335 [− 0.448; − 0.222]). There was no significant interaction of group and image type (χ^2^_(1)_ = 0.028, p = 0.867, d_t_ = − 0.013 [− 0.168; 0.141], see Fig. 2a).

### Persistence Task

Belonging to group CON did not affect persistence time (χ^2^_(1)_ = 2.133, p = 0.14, d_t_ = − 0.290 [− 0.662; 0.082]). Social stimuli increased persistence time by about 0.089 s (se = 0.044 s) (χ^2^_(1)_ = 11.689, d_t_ = 0.093 [0.002; 0.185], p < 0.001). There was no significant interaction of group and image type (χ^2^_(1)_ = 0.199, p = 0.66, d_t_ = 0.028 [− 0.096; 0.153], see Fig. 2c).

### Exploratory Analysis

The introduction of a three-way interaction of response subset*image type*group significantly improved the model fit (χ^2^_(1)_ = 3.862, p = 0.049, d_t_ = − 0.294 [0.001; 0.587]) compared to all models including single interactions with an estimate (group CON, late, social) of 0.307 s (se = 0.16 s). In social stimuli, group differences appeared larger in early compared to late responses while in non-social stimuli, group differences appeared smaller for early compared to late responses (Fig. 2b). Separate post-hoc analyses of the early and late response subsets revealed no significant interactions of diagnostic group*image type (subset early χ^2^_(1)_ = 2.369, p = 0.12, subset late χ^2^_(1)_ = 1.634, p = 0.20).

**Fig. 3:**
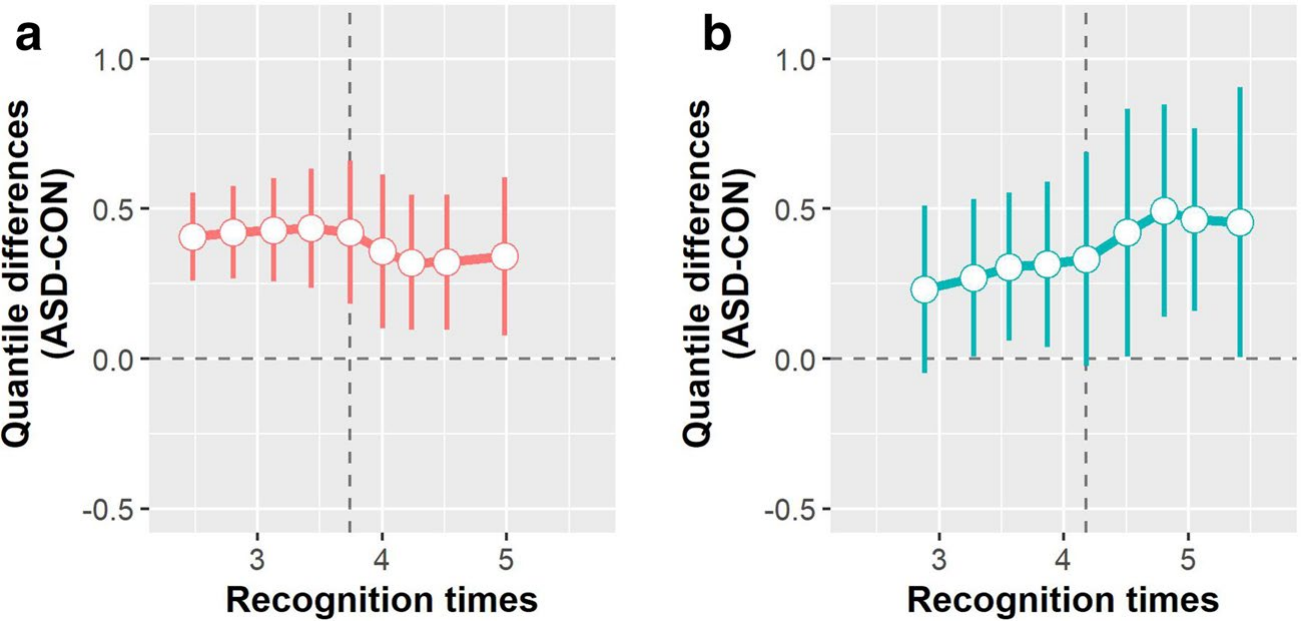
Quantile-wise comparison of averaged recognition times for all social and non-social stimuli between diagnostic groups (shift function). The difference ASD–CON is depicted along the y-axis for each decile (white disks), as a function of ASD deciles. For each decile difference, the vertical line indicates its 95% bootstrap confidence interval. When a confidence interval does not include zero, the difference is considered significant in a frequentist sense. **a** Shift function for the emergence of social stimuli. **b** Shift function for the emergence of non-social stimuli

However, a quantile-wise analysis (Fig. 3) elucidates the changes in the difference between diagnostic groups depending on the complexity of the stimuli, i.e., for early vs. late recognized images. These changes seem to have opposite directions for social and non-social stimuli with higher complexity leading to a decrease of group differences for social stimuli and an increase for non-social stimuli.

## Discussion

In the current study we compared the performance of persons with and without ASD differentiating between social and non-social stimuli in two different perceptual tasks. Social stimuli were recognized significantly faster than non-social stimuli by both groups, as described by previous reports of preferential processing of social over non-social stimuli (Bruce et al., 1991; Kikuchi et al., 2009; Lehky, 2000; Ro et al., , 2001, 2007). This is also in accordance with neuroscientific evidence of pathways specialized for the processing of socially relevant information (Alcalá-López et al., 2018; Nummenmaa & Calder, 2009).

Participants with ASD were generally slower to respond to emerging stimuli. This delay to recognize social and non-social images might be explained by the fact that global, semantic processing is essential for our task. Individuals with ASD have been reported to perceive global order or the gist of a scene slower compared to CON (Van der Hallen et al., 2015; Vanmarcke et al., 2016a, 2016b). This could also be interpreted in the context of difficulties in persons with ASD in gestalt processing (Bölte et al., 2007; Brosnan et al., 2004; Gowen et al., 2020). Relatedly, the increased response times observed in individuals with ASD may correspond to differences in central coherence (Brock et al., 2002; Frith, 2003; Happé, 1999). Weak central coherence theory states that behavioral symptoms of ASD are exhibited due to the comparatively low integration of complex sensory information, with more pronounced focus on detail than on context. We instructed participants to perform a task which required persistent integration of changing stimuli. Considering the assumed differences in information integration for persons with ASD, solving this task would require more time as compared to persons without ASD. The latter group would perceive the changing picture as changing globally, with the presented picture as a continuous and synchronous, stable percept. Conversely, perceptual processing in the ASD group would be directed at the pixel changes more locally, merging these changes into a coherent percept only afterwards; hence requiring more time. These considerations relate to recent theory on the importance of time and temporal processing in the emergence of autistic symptoms in persons with ASD, when faced with interactive and social situations (Bloch et al., 2019; Hohwy et al., 2016; Vogel et al., 2019). This idea is further corroborated by neither findings of general impairments in reaction times in persons with ASD (Ferraro, 2016), nor findings of visual perception being slower or less accurate than in persons without ASD per se (Remington et al., 2009; Vanmarcke et al., 2016b).

A potential caveat of the recognition task is that social stimuli (depicting people) might be less heterogeneous than non-social images and are more frequently presented. This could have biased attention and could have facilitated the recognition of social stimuli. However, in the persistence task, all stimuli were already familiar and visible in the beginning of the trial, thus minimizing attention and homogeneity biases. Here, again, social stimuli seem to have been recognizable baring higher levels of noise (i.e. social stimuli were recognizable longer). Thus, the persistence task further corroborates that social stimuli are indeed processed preferentially. Furthermore, although a recent meta-analysis suggests no general impairment, mixed findings exist regarding reaction times in ASD (Ferraro, 2016). Therefore, results from the persistence task also speak against a mere reaction time bias in ASD in the recognition task.

Results in the persistence task did not differ between diagnostic groups and thus did not indicate any general tendency towards response delay or increased avoidance of uncertainty in ASD. We also have no reason to assume that social scenes compared to non-social scenes persisted longer in control subjects compared to subjects with ASD. One explanation could be that control participants were superior in the automatic processing of social stimuli and that this was more decisive in the recognition task. Implicit and automatic features of social cognition have repeatedly been emphasized (Bargh, 1994; Choi et al., 2005) and it seems that these features are especially difficult for persons with ASD (Eigsti, 2013; McIntosh et al., 2006).

We did not find specific effects of social stimuli in persons with ASD. This stands in contrast to previous studies, reporting decreased attention for social stimuli in ASD. However, recently, the account of a general reduction in attention to social stimuli in ASD has been challenged (Guillon et al., 2016). Instead, it was argued that impairments in attention towards social stimuli in ASD seem to be dependent on the complexity and contextual factors of stimuli (Chita-Tegmark, 2016; Guillon et al., 2014; Hamilton, 2016). Following this proposal, we investigated in an exploratory analysis the effect of stimulus complexity on recognition times. Group differences in social images seemed to be generally more pronounced in low complexity stimuli and decreased with complexity while the opposite was true for non-social stimuli. Thus, the relationship between the social character and the complexity in the recognition of pictures in ASD requires further investigations.

In future studies, an in-depth analysis of the characteristics responsible for early or late recognition could enable a discussion in current frameworks, e.g. weak central coherence theory (Happé & Booth, 2008; Happé & Frith, 2006; Koldewyn et al., 2013) or alternatively the enhanced perceptual functioning account (Mottron et al., 2006). In particular, since recent meta-analyses have questioned general deficits in ASD global processing (Van der Hallen et al., 2015) but find delayed processing or a specificity in social images (Vanmarcke et al., 2016b), more detailed studies are required.

We did not find influences of age or gender in our tasks, which is in line with several meta-analyses that find a stable social attention bias between persons with ASD and control persons across ages and gender (Chita-Tegmark, 2016; Frazier et al., 2017). However, there is also evidence that the social attention bias might be more pronounced and homogenous in children in the age range of 0.5–5 years (Guillon et al., 2014) and that results for children/adolescents (age range of 5–18 years) and adults are much more heterogeneous. It is very possible that persons with ASD learn to compensate for a social attention bias and that this task would have very different results in younger children.

In summary, our results showed that control subjects were faster in recognizing images compared to subjects with ASD. Both groups recognized social scenes, i.e., images containing persons, faster than images containing animals or objects. Contrary to our hypothesis, we did not find a general impairment in the recognition of social scenes in persons with ASD compared to control subjects. However, exploratory analyses suggested that the complexity of the depicted scenes might be experienced differently by subjects with and without ASD in social and non-social stimuli.

## Limitations

The two diagnostic groups differ significantly in age by about 5 years (sd = 9 years). However, separate analyses of the influence of age and gender did not point towards significant influences on the tasks. BDI scores were slightly higher in the ASD group compared to controls but none of these were above a clinically relevant threshold and the validity of these scores might be limited in persons with ASD (Cassidy et al., 2018). The generalizability of our findings might be limited, since our sample sizes were rather small and our sample of adult persons with ASD was very well educated and highly functioning. In other age groups or clinical populations, results might be different.
